# Subcritical water extraction of bioactive compounds from *Orostachys japonicus* A. Berger (Crassulaceae)

**DOI:** 10.1038/s41598-020-67508-2

**Published:** 2020-07-02

**Authors:** Min-Jung Ko, Hwa-Hyun Nam, Myong-Soo Chung

**Affiliations:** 1 Department of Food Science and Biotechnology, Global K-Food Research Center, Hankyong National University, Anseong-si, 17579 South Korea; 2 Department of Food Science and Engineering, Ewha Womans University, Seoul, 03760 South Korea

**Keywords:** Plant sciences, Plant ecology

## Abstract

Subcritical-water extraction is an ecofriendly method for extracting antioxidant compounds only using water. The Subcritical-water extraction was employed for the extraction of bioactive compounds from *Orostachys japonicus* known as rock pine by investigating the use of various temperatures (110–260 °C) and extraction times (5–20 min). The Subcritical-water extraction condition at 220 °C for 15 min; the total phenolics content (39.9 ± 4.1 mg/g), flavonoids content (11.4 ± 0.6 mg/g), and antioxidant activities (90.3 ± 2.2%, 96.0 ± 2.9%, and 662.4 ± 17.2 mg/g) of Subcritical-water extract were higher under this condition than for extraction with either methanol or ethanol. Triterpene saponins were observed only in subcritical-water extraction condition at 220 °C for 15 min. Further, some of its phenolic constituents; gallic acid, quercetin, and kaempferol were quantified by high performance liquid chromatography. Subcritical-water extraction is an effective method for extracting valuable bioactive compounds from *Orostachys japonicus*.

## Introduction

*Orostachys japonicus* A. Berger (Crassulaceae), which is often referred to as rock pine because its shape resembles that of a pine-tree cone, is a perennial herb whose main habitat is on the surface of mountain rocks in South Korea and Japan. The extract of *Orostachys japonicus* (OJ) has been utilized as a medicine in South Korea for treating various diseases including fever, gingivitis, metritis, coagulation, intoxication, and cancer^1^. This is because OJ contains many biologically active compounds such as polyphenolic compounds including flavonoids, and triterpenes including saponins. Phenolics are compounds in which a hydroxyl group is attached to benzene, and they include flavonoids^2^, which are widely distributed in plants and are a component of the human diet^3^. OJ contains the flavonoids quercetin, kaempferol, and gallic acid^1^. Triterpenes are composed of three terpene units with the molecular formula C_30_H_48_, and they may also be thought of as consisting of six isoprene units. Their biological activities include antioxidant activity, radical-scavenging effects, antiallergic activity, and cardioprotective effects^4^.

Previous studies showed that there are significant differences in biological activities of extracts of OJ between fractionation solvents. The methanol extract showed the highest antibacterial activity among extraction solvents such as methanol, ethanol, and hot water extract^5^, and the highest cytotoxic effects result was in ethylacetate fractionated extract among hexane, methylene chloride, ehtylacetate, and butyl alcohol depending on the applied solvent polarity from OJ^6^. Also, OJ aqueous extract exhibited antioxidant effects, including DPPH radicals, and might play an immunostimulatory activity^7^.

Although methanol and ethylacetate extracts showed high biological activities, its utilization for food is limited due to toxicity and long extraction times of more than 2 h. Subcritical-water extraction (SWE) is a relatively new technique for extracting less-polar compounds using only water for short extraction time in 30 min^8^. Subcritical water is maintained in a liquid state under high pressure at a temperature between 100 and 374 °C^9^. Water at a higher temperature has a lower dielectric constant, which weakens the hydrogen bonds and makes subcritical water more similar to less-polar organic solvents such as methanol and ethanol^10^. The solubility of less polar phenolics increased when the temperature of subcritical water was increased. Our previous studies, the selective extraction of the flavonoid quercetin was possible using subcritical water, and that the yield of extracts was higher when using subcritical water than when using ethanol, methanol, or hot water^11^. SWE is an environmentally friendly and efficient extraction method that does not require the use of an organic solvent to extract phenolics and flavonoids. There has been an increasing interest in the use of ecofriendly technologies. SWE can provide high biological activities of extracts while precluding any toxicity solvents.

The objective of this study was to determine the optimum extraction temperature (110–260 °C) and extraction time (5–20 min) for extracting bioactive components (phenolics, flavonoids) from OJ in both laboratory- and pilot-scale SWE experiments. The effects of the SWE conditions on the antioxidant activity (DPPH-radical-scavenging activity, ABTS-radical-scavenging activity, FRAP assay) of the extracts were also assessed. SWE yields and antioxidant activities were compare with those obtained among solvents involving methanol, and ethanol. Especially, the thermal stability of the bioactive compounds in OJ at the extraction temperature higher than 200 °C was confirmed in this study.

## Materials and methods

### Sample preparation

OJ samples were purchased from the South Korea bawisol Cooperative as a hot-air-dried powder. The moisture content of the samples was 7.6 ± 0.1%, as measured using an infrared moisture balance (FD 610, Kett, Tokyo). The samples were stored at 4 °C.

### Laboratory-scale SWE

**Figure 1:**
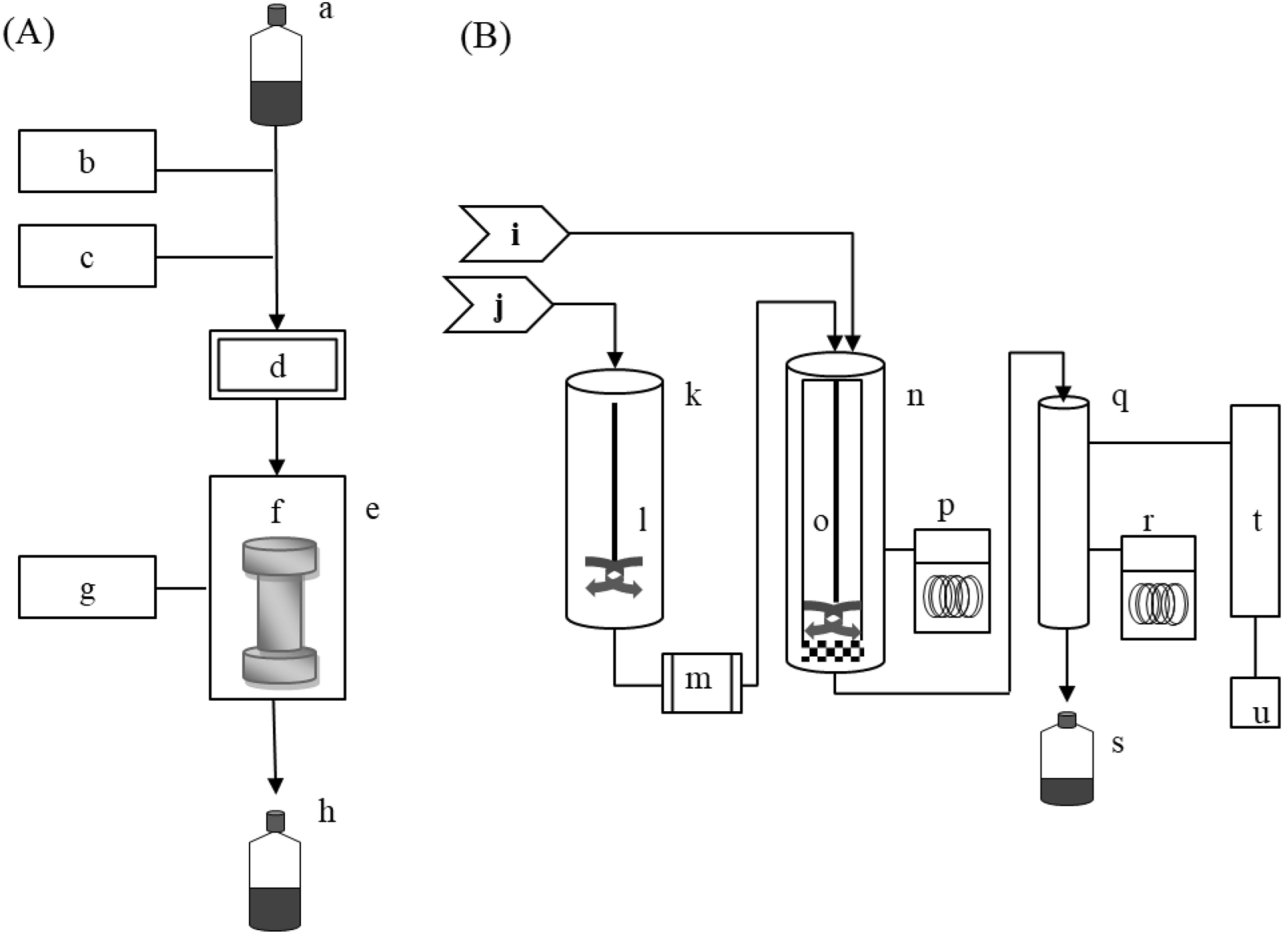
Schematic diagrams of the laboratory-scale (**A**) and pilot-scale (**B**) SWE systems, showing the solvent bottle (a), pump (b), nitrogen gas (c), pressure transducer (d), oven (e), extraction cell (f), temperature controller (g), collection bottle (h), nitrogen gas (i), water supply (j), preheater (k), stirring bar (l), pump (m), extractor (n), stirring bar (o), cooling system (p), collector (q), cooling system (r), collection bottle (s), condenser (t), and computer control system (u).

Laboratory-scale SWE was performed using an accelerated solvent extractor (ASE 350, Dionex, Sunnyvale, CA, USA) with purified water and the following procedures: The sample (1 g) plus diatomaceous earth (3 g; ASE Prep DE, Dionex) were extracted with water in a stainless-steel extraction cell having a volume of 22 mL and containing a cellulose filter (30 mm; Whatman, Maidstone, UK) on the bottom. The extraction process is shown in Fig. 1A. The extraction cell (f) was placed in an oven (e), filled with water for 30 s, and then heated under high pressure (≈ 10 MPa) for the required extraction time. The extract was then purged for 1 min at about 10 MPa, often with nitrogen gas, and the residual pressure was then released. The final extract was collected in a collection bottle (h). The experiment was performed under 24 conditions that combined 6 extraction temperatures (110 °C, 130 °C, 150 °C, 170 °C, 190 °C, and 200 °C) and four extraction times (5, 10, 15, and 20 min). All of the aqueous extracts were dried in a freeze-dryer for 24 h (Ilshin, Gyeonggi-do, Korea).

### Pilot-scale SWE

The pilot-scale SWE system (Fig. 1B) was developed to scale up the laboratory-scale system approximately 80-fold. The pilot system included the following components: a preheater (k) with an agitator (l), an extractor (n) with an agitator (o), a collector (q), a stainless-steel cell, and ancillary piping systems (R-401, Reaction Engineering, Gyeonggi-do, Korea). A computer-based system with control software (R-SCS, Reaction Engineering) was used to operate the pilot-scale SWE system. All extractions were carried out using an 8-L stainless-steel cell (143 mm i.d. × 520 mm long). The tubular extraction cell containing a cellulose filter on the bottom was filled with the sample (30 g) and then placed in the extractor, followed by the transfer of 0.9 L of preheated water at 60–80 °C into the extractor. This was then heated to the designated temperature and maintained at that temperature for the designated extraction time. After the extraction time, the extract (about 0.7 L) was transferred to the collector automatically and the residue in the extractor was recovered with the aid of nitrogen gas. Aliquots (30 mL) of the collected extracts were freeze-dried for 24 h and stored at 4 °C for use in subsequent experiments. Since the maximum operating temperature of our laboratory-scale SWE apparatus (ASE 350, Dionex) is 200 °C, however, we could try to conduct extraction operations at higher temperature than 200 °C using a self-designed pilot-scale SWE (up to 300 °C). This experiment using the pilot-scale SWE system was necessary to investigate the bioactive compounds yield above 200 °C, which could not be applied in the laboratory-scale system. In the present study, the extraction temperatures (150, 170, 190, 200, 220, 240, and 260 °C) and time (15 min) were used as extraction parameters by the pilot-scale SWE system.

### Pretreatment of sample extracts

All of the aqueous extracts were dried in a freeze-dryer for 24 h. Freeze-dried extract (5 mg) obtained by laboratory-scale and pilot-scale SWE was dissolved in 5 mL of methanol and then sonicated for 2 h by filtration through a 0.45 microm PVDF filter (Whatman, Maidstone, United Kingdom) to measure the phenolics content, and antioxidant activity including DPPH free-radical-scavenging activity, ABTS-radical-scavenging activity, and FRAP assay.

### Triterpene identification

The extracts were qualitatively analyzed triterpene by high-performance liquid chromatography mass spectrometry (HPLC–MS; 1,290 series–6,530 Accurate-Mass Q-TOF, Agilent Technologies, Waldbronn, Germany) with an Extend C18 column (2.1 mm × 50 mm, 1.8-μm pore size; Agilent Technologies), which is equipped with an electrospray ionization source (ESI). The analysis parameters were set using negative ion modes, with spectra acquired for mass-to-charge values of m/z = 50–3,000. HPLC analysis was applied by modifying the previous method^4^. HPLC conditions are as follows: solvent A, 0.2% acetic acid in water; solvent B, acetronitrile; 0–15 min (35%–40% B), 15–45 min (40%–60% B), 45–55 min (60%–80% B), 55–56 min (80%–35% B), 56–60 min (35% B); flow rate, 1.0 mL/min, and detection was performed at 280 nm.

### Phenolics content analysis

The gallic acid was analyzed by HPLC^12^. HPLC was performed using an Agilent system (1,260 series, Agilent Technologies, Santa Clara, USA) with a Zorbax C18 Column (4.6 mm × 150 mm, 5 μm pore size; Agilent Technologies). For analyzing gallic acid, the mobile phase used for analysis comprised 25% methanol in 1% acetic acid at a flow rate 0.75 mL/min. Twenty-microliter aliquots were injected into the HPLC system, and a detection was performed at 280 nm.

The total phenolics content was determined using the modified Folin-Ciocalteu reagent method. Briefly, 0.1 mL of pretreated extracts was mixed with 2 mL of 2% NA_2_CO_3_, vortexed for 3 min, and then 0.1 mL of 50% Folin-Ciocalteu reagent was added to the mixture. After 30 min at room temperature, the absorbance was measured at 700 nm using a spectrophotometer (Evolution 200, Thermo Scientific, Waltham, MA, USA). Gallic acid was used as a standard to obtain a calibration curve. The total phenolics content was expressed in milligrams of gallic acid equivalent (GAE) per gram fresh weight of OJ (mg GAE/g).

### Flavonoids content analysis

The contents of flavonoids such as quercetin and kamepferol were analyzed by HPLC^13^. HPLC was performed using an Agilent system (1,260 series, Agilent Technologies, Santa Clara, USA) with a Zorbax C18 Column (4.6 mm × 150 mm, 5 μm pore size; Agilent Technologies). For analyzing quercetin and kaempferol, the mobile phase used for analysis comprised 1% acetic acid in water (solvent A) and acetronitrile (solvent B) at a flow rate 1 mL/min with an initial ratio of 90%:10% (A:B). The ratio of solvent B was changed as follows: to 15% over 16 min, to 50% over 2 min, and to 10% over 5 min. Twenty-microliter aliquots were injected into the HPLC system, and a detection was performed at 370 nm.

The total flavonoids content was measured using the aluminum chloride colorimetric method^14^. A 0.5-mL sample of pretreated extract was mixed with 1.5 mL of 95% ethanol, and then 0.1 mL of 10% aluminum chloride and 1 M potassium acetate were added. The absorbance of the mixture was measured at 415 nm using a spectrometer after a reaction time of 30 min at room temperature. The standard curve was obtained using quercetin. The total flavonoids content was expressed in milligrams of quercetin equivalent (QE) per gram dry weight of OJ (mg QE/g).

### DPPH-radical-scavenging activity

The 2,2-diphenyl-1-picrylhydrazyl (DPPH)-radical-scavenging capacity of each extract was determined^15^. DPPH radicals exhibit an absorption maximum at 515 nm, which disappears upon reduction by an antioxidant compound. The DPPH^·^ solution in methanol (6 × 10^–5^ M) was prepared daily, and 3 mL of this solution was mixed with 100 μL of methanolic solutions of plant extracts. The samples were incubated for 20 min at 37 °C in a water bath, and then the decrease in the absorbance at 515 nm was measured. A blank sample containing 100 μL of methanol in the DPPH^·^ solution was prepared daily, and its absorbance was measured. The experiment was carried out in triplicate. The radical-scavenging activity was calculated using the following formula.

1 $$ {\text{DPPH-radical-scavenging activity}}\;\left( \% \right) = \left[ {\left( {{\text{AB}}{-}{\text{AE}}} \right)/{\text{AB}}} \right] \times 100 $$

where AB is the absorbance of the blank sample and AE is the absorbance of the plant extract.

### ABTS-radical-scavenging activity

The free-radical-scavenging capacity of extracts was also studied using the ABTS (2,2′-azino-bis-3-ethylbenzthiazoline-6-sulphonic acid)-radical-cation (ABTS^+·^) decolorization assay, which is based on the reduction of ABTS^+·^ radicals by antioxidants in the tested extracts. ABTS was dissolved in deionized water to a concentration of 7 mM. ABTS^+·^ was produced by reacting ABTS solution with potassium persulfate with a final concentration of 2.45 mM and allowing the mixture to stand at room temperature for 12–16 h before use. For this study the ABTS^+·^ solution was diluted in deionized water or ethanol so that the absorbance at 734 nm was 0.7. An appropriate solvent blank reading was taken. After adding 100 μL of aqueous or ethanolic plant extract solutions (according to the solubility) to 3 mL of the ABTS^+·^ solution, the absorbance was measured at 30 °C and 10 min after the initial mixing. All solutions were used on the day of preparation, and all measurements were made in triplicate. The percentage of radical scavenging activity of ABTS^+·^ was calculated using the above formula.

### FRAP assay

The ferric reducing antioxidant power (FRAP) assay was implemented^16^. A 300 mM acetate buffer was made using 3.1 g of C_2_H_3_Na_2_·3H_2_O and 16 mL of C_2_H_4_O_2_. The fresh working FRAP reagent was made by mixing acetate buffer, 10 mM tripyridyltriazine (TPTZ) in 40 mM HCl, and 20 mM FeCl_3_·6H_2_O solution at a ratio of 10:1:1. The working FRAP reagent was preheated at 37 °C before use. Sample aliquots (10 μL) were reacted with 300 μL of the working FRAP reagent on a 96-well plate for 30 min in the dark at 37 °C. The absorbance of the colored product (ferrous TPTZ complex) was measured at 593 nm using a microplate reader (BNR 05606, VersaMax, Sunnyvale, CA). The standard curve was linear between 0.000977 and 0.5 mg/mL trolox. The results are expressed in milligrams per milliliter trolox equivalent (TE) per gram fresh weight of OJ (mg TE/g).

### Chemicals

HPLC solvents including methanol (≥ 99.9%), water (≥ 100%), and acetonitrile (≥ 99.9%) were purchased from J.T. Baker (Phillipsburg, NJ, USA). Folin-Ciocalteu reagent, gallic acid (3,4,5-trihydroxybenzoic acid, ≥ 99%), kaempferol (≥ 97%), quercetin (≥ 95%), DPPH (≥ 100%), ABTS^·+^ (≥ 100%), trolox (≥ 97%) were purchased from Sigma-Aldrich (St. Louis, MO, USA). Methanol (≥ 99%; Duksan, Gyeonggi-do, Korea), Na_2_CO_3_ (≥ 99.9%; Duksan), ethanol (≥ 99.9%; Samchun, Pyeongtaek, Gyeonggi-do, Korea), aluminum chloride (98%; Junsei, Japan), and potassium acetate (≥ 99%; Duksan) were also used.

### Data analysis

The content was calculated from the calibration curve of standard compounds. The optimum SWE conditions from OJ were chosen based on the highest content or activity. All data are given as mean ± SD values of three measurements. One-way analysis of variance with Duncan test, and Pearson correlation analysis were performed using SPSS (version 24.0, IBM SPSS, Chicago, IL, USA).

## Results and discussion

### Triterpene saponins

**Figure 2:**
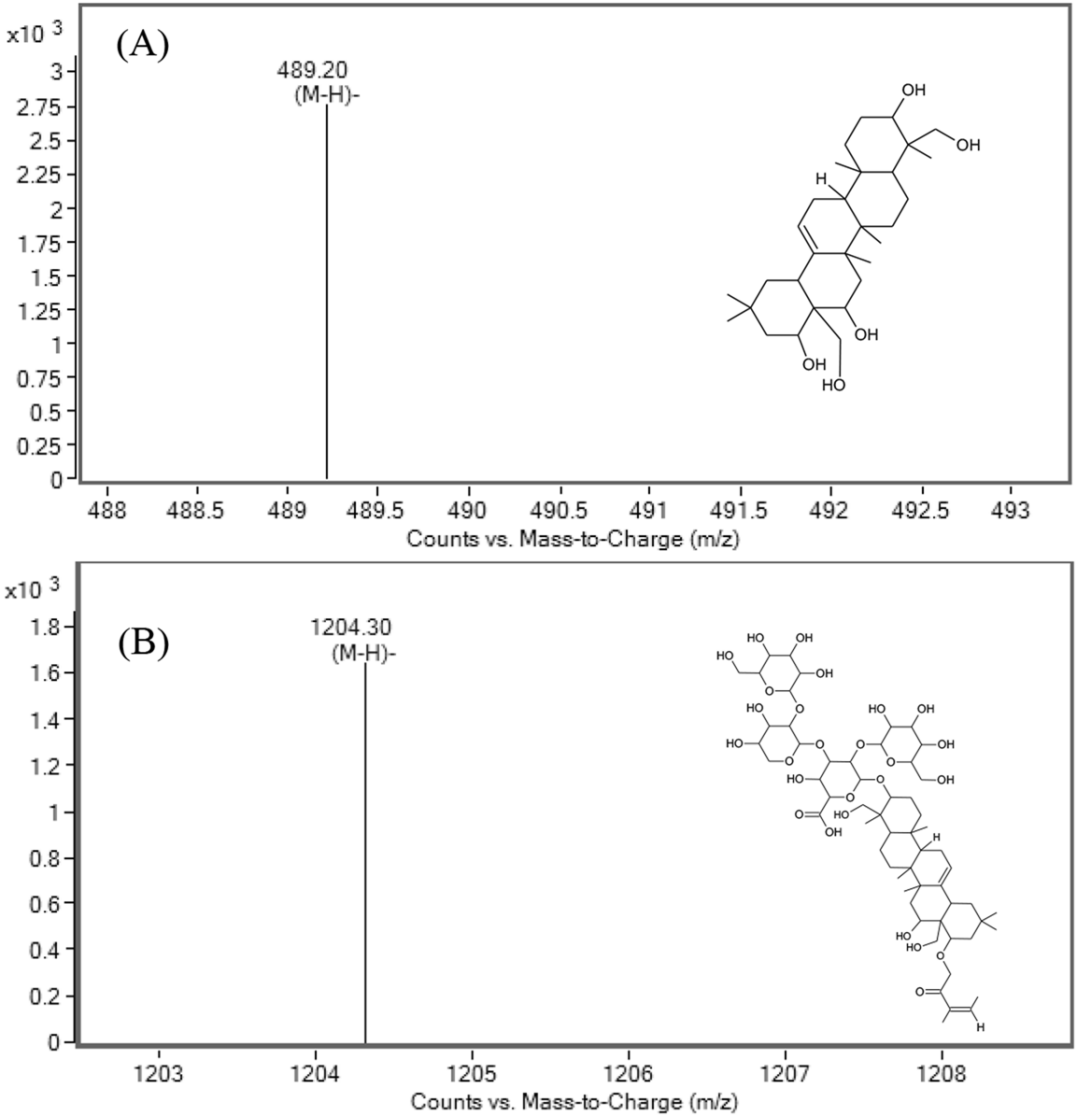
Characteristics of triterpene saponins extract from OJ.

We analyzed qualitatively the triterpene saponins of OJ extracts for SWE times of 15 min at temperatures of 110, 150, and 220 °C by HPLC/ES-MS. The triterpene saponins that could be detected by the HPLC–ESI–MS systems are shown in Fig. 2. The compounds were identified as Camelliagenin (C_30_H_50_O_5,_ Molecular weight: 490.72), and Camellia Saponin (C_58_H_92_O_26,_ Molecular weight: 1,205.34) based on the MS spectral data. Camelliagenin, and Camellia Saponin m/z values of 489. 20 [M-H]^−^, and 1,204.30 [M-H]^−^ in the negative ionization condition were observed, respectively. These compounds are mainly saponins from Camelia oleifera Abel, and it was reported that those exhibit anticancer activity^17^. These compounds were observed only in SWE condition of 220 °C/15 min, not detected in other conditions such as 110 °C and 150 °C of SWE extracts. It also showed that the highest antioxidant activities values such as DPPH, ABTS, and FRAP assay in this condition (220 °C/15 min). SWE extracts seemed to affect the antioxidant activity due to the presence of not only phenolics including flavonoids but also triterpene saponins. The solubility of a bioactive compound in subcritical water is influenced by the chemical structure of the solute, and the complex interactions between the solvent of water and the solute. The solubility of hydrophobic organic compounds in subcritical water is dependent upon the degree of conjugation of the aromatic rings, and the presence of different side-groups around the hydrophobic organic compounds^18^. As the greater the aromatic hydrocarbons rings such as triterpene saponins, the higher the solubility at higher temperature (220 °C) of subcritical water. The thermal stability of process of extracting triterpene saponins was observed up to 210 or 220 °C by thermogravimetric and derivative thermogravimetric analysis, indicating stability of triterpene saponins in the subcritical water^19^. The thermal decomposition process showed a sharp peak at approximately 350 °C in the previous studies^20^.

### Phenolics content

The gallic acid (trihydroxybenzoic acid) is a type of phenolic acid frequently used for a standard for determining the total phenolics content analysis. The gallic acid content of OJ extracts increased with the extraction temperature and extraction time on subcritical water conditions. The gallic acid content was maximal for extraction at 190 °C for 15 min (6.3 ± 0.2 mg/g OJ) by HPLC analysis.

**Figure 3:**
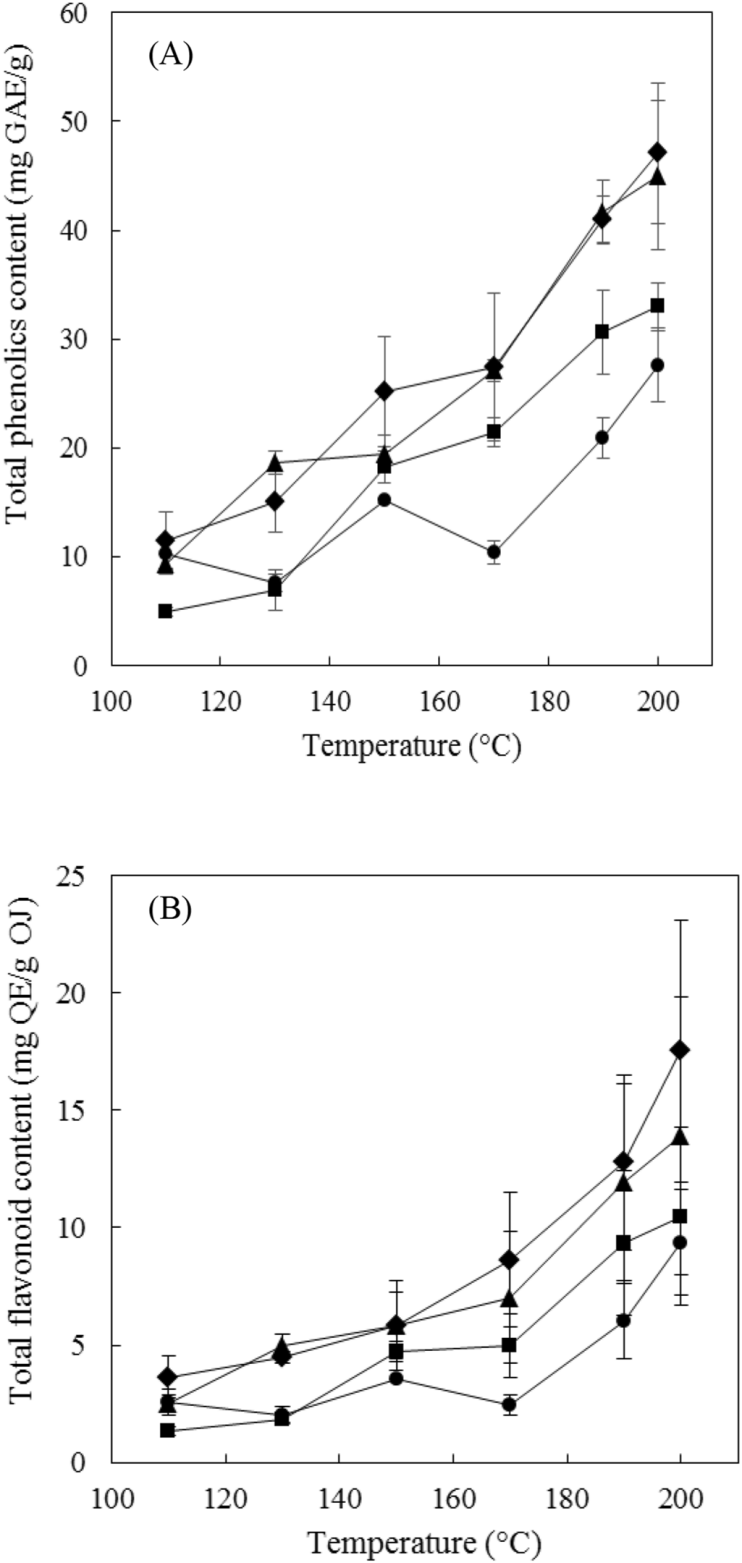
Effects of the extraction temperature of SWE on the total phenolics content (**A**) and the flavonoids content (**B**) in OJ for extraction times of 5 min (filled circle), 10 min (filled square), 15 min (filled triangle), and 20 min (filled diamond). The data are mean and SD values (*n* = 3).

As shown in Fig. 3A, the total phenolics content of OJ extracts increased with the extraction temperature and extraction time. The reduction in the dielectric constant of subcritical water as its temperature increases results in more nonpolar phenolics being extracted^21^. The SWE of nonpolar phenolics was affected by physical property such as the dielectric constant of water, which decrease with increasing temperature. A low dielectric constant means that the water molecules their hydrogen bonds are broken. Disruption of the hydrogen bond of water increases the solubility of nonpolar solutes. The total phenolics content of the extracts was greater at 200 °C than at 100 °C, which is consistent with the findings^22,23^. A high thermal stability of phenolics in high-temperature subcritical water was also reported^24^. The highest total phenolics content of 47.1 ± 6.5 mg GAE/g was obtained at 200 °C for 20 min using laboratory-scale SWE. The maximum extraction temperature of our laboratory-scale SWE apparatus (ASE 350, Dionex) is 200 °C, and so we could only perform extraction operations at higher temperatures (up to 240 °C) using the pilot-scale apparatus (R-401, Reaction Engineering). The analysis results in Table 1 indicate that the total phenolics content at an extraction temperature of 220 °C was optimally 39.9 ± 4.1 mg GAE/g when using pilot-scale SWE. The phenolics were destroyed at higher temperatures up to 240 °C.

**Table 1:** Effects of the extraction temperature of SWE on the total phenolics content, flavonoids content, and antioxidant activities in OJ for an extraction time of 15 min. Means in a column followed by same superscript letters are not significantly different according to Duncan’s test at *p* < 0.05.

SWE scale	Extraction temperature (°C)	Extraction time (min)	Total phenolics content (mg GAE/g)	Total flavonoids content (mg QE/g)	DPPH-radical-scavenging ability (%)	ABTS-radical-scavenging ability (%)	FRAP antioxidant activity (mg TE/g)
Laboratory	110	15	9.4 ± 11.0^a^	2.5 ± 0.4^a^	65.8 ± 4.5^a^	27.6 ± 0.7^a^	148.0 ± 14.3^a^
	130	15	18.6 ± 1.1^b^	5.0 ± 0.5^bc^	82.4 ± 1.1^cd^	41.6 ± 6.7^b^	150.5 ± 12.6^a^
Pilot	150	15	22.1 ± 5.8^bc^	4.1 ± 0.4^b^	71.5 ± 4.0^b^	49.6 ± 3.9^c^	246.5 ± 18.1^ab^
	170	15	26.2 ± 4.3^c^	4.5 ± 0.5^bc^	79.9 ± 1.1^c^	67.6 ± 4.1^d^	348.3 ± 14.6^b^
	190	15	34.5 ± 3.7^de^	5.3 ± 0.5^bc^	84.8 ± 1.9^d^	80.1 ± 1.6^e^	492.5 ± 8.9^c^
	200	15	32.4 ± 1.6^d^	9.7 ± 0.9^d^	89.4 ± 0.8^e^	86.6 ± 3.7^ef^	598.1 ± 21.6^cd^
	220	15	39.9 ± 4.1^e^	11.4 ± 0.6^e^	90.3 ± 2.2^e^	96.0 ± 2.9^g^	662.4 ± 17.2^d^
	240	15	33.6 ± 4.2^de^	5.5 ± 1.2^c^	89.4 ± 0.2^e^	89.8 ± 3.2^fg^	520.8 ± 14.7^c^

The data are mean ± SD values (*n* = 3).

### Flavonoids content

We analyzed the quercetin, and kaempferol content of OJ extracts for SWE conditions by HPLC analysis. The quercetin is usually used for a standard for determining the total flavonoids content analysis. The quercetin, and kaempferol content of OJ extracts increased with the extraction temperature and extraction time using SWE. The quercetin (853.2 ± 0.6 mg/kg OJ), and kaempferol (61.0 ± 0.3 mg/kg OJ) content was maximal for extraction at 170 °C for 15 min, and 190 °C for 15 min, respectively.

Figure 3B shows that the total flavonoids content of the extracts obtained using laboratory scale SWE increased with the extraction temperature and time, which was similar to the results for the total phenolics content. The total flavonoids content was particularly affected by the extraction temperature, increasing significantly between 170 and 190 °C. The highest total flavonoids content of 17.5 ± 5.6 mg QE/g was obtained at 200 °C for 20 min using laboratory-scale SWE. Flavonoids such as gallic acid, kaempferol, quercetin, kaempferol 3-O-β-d-glucoside, kaempferol 3-O-β-d-galactoside, and quercetin 3-O-β-d-glucoside in water extracts from OJ^25^. These findings suggest that most of the phenolics in an OJ extract at higher temperatures are stable flavonoids. These flavonoids might have affected the present results because quercetin and kaempferol are stable in subcritical water at high temperature^14^.

The total flavonoids content at an extraction temperature of 220 °C of 11.38 ± 0.59 mg QE/g indicated optimal pilot-scale SWE. The flavonoids were degraded at higher temperatures up to 240 °C. Hydrocarbons such as flavonoids were also found to degrade rapidly in pressurized hot water at 250 °C^26^. Our previous studies showed that there was no significant difference in the extraction yields among various pressures in SWE^27,28^. Dielectric constant also indicated that the solubility of water does not significantly change as the pressure below 200 bar.

### DPPH-radical-scavenging activity

The DPPH assay is a mixed method based on both hydrogen-atom transfer and single-electron transfer^29^. The DPPH-radical-scavenging activity is measured as the number of DPPH· that decrease as in the following reaction^15^.

2 $$ {\text{DPPH}}^{\cdot} \, + {\text{ AH}} \to {\text{DPPHH}} + {\text{A}}^{\cdot} $$

**Figure 4:**
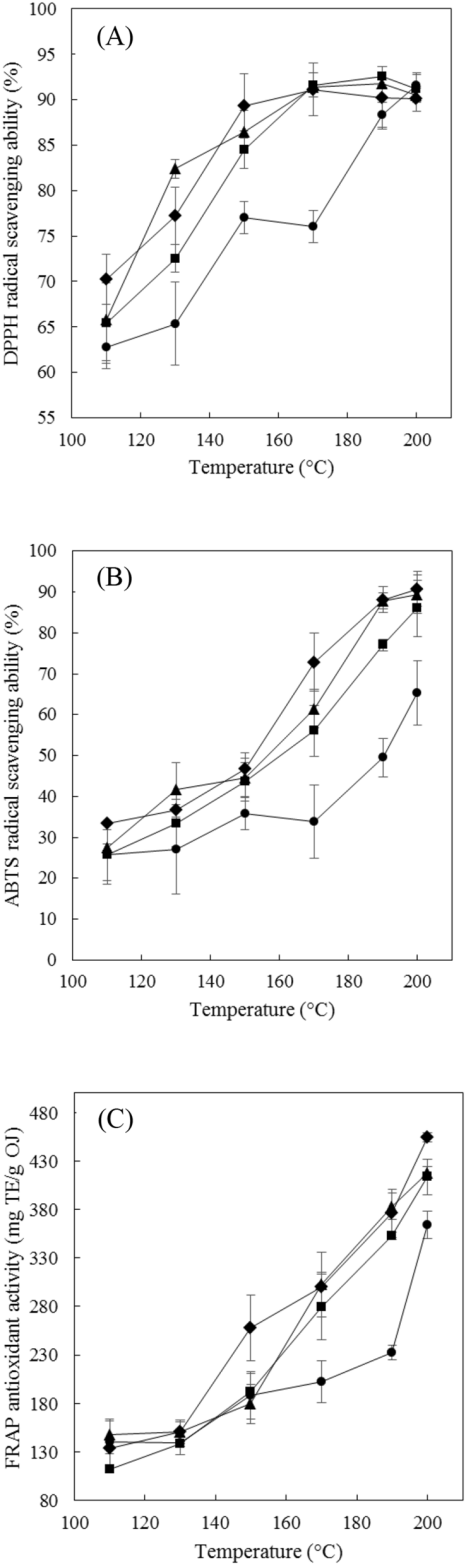
Effects of the extraction temperature of SWE on DPPH-radical-scavenging ability (**A**), ABTS-radical-scavenging ability (**B**), and FRAP (**C**) in OJ for extraction times of 5 min (filled circle), 10 min (filled square), 15 min (filled triangle), and 20 min (filled diamond). The data are mean and SD values (*n* = 3).

The DPPH-radical-scavenging activity increased with the extraction temperature and extraction time (Fig. 4A). The highest scavenging activity of 91.8 ± 0.4% was obtained at 190 °C and 15 min in laboratory-scale SWE. This temperature is lower than the optimum temperature (200 °C) for maximizing the yield of phenolics and flavonoids. This result is similar to our previous finding of SWE extracts affecting the antioxidant activity at higher temperatures around 200 °C due to the presence of both flavonoids and nonflavonoids^21^. Moreover, the antioxidant capacities as measured by the DPPH assay were partly correlated with the phenolics and flavonoids contents in SWE^21^.

The analysis results for the DPPH assay indicated that an extraction temperature of 220 °C was optimal (90.3 ± 2.2%) when using pilot-scale SWE (Table 1). The DPPH assay was decreased at higher temperatures up to 240 °C.

### ABTS-radical-scavenging activity

The ABTS assay is also a mixed method using hydrogen-atom transfer and single-electron transfer. ABTS^·+^ radical cations were generated from ABTS^30^, and the ABTS-radical-scavenging activity is measured based on the decrease in ABTS^·+^ in the following reaction^31^.

3 $$ {\text{ABTS}}^{\cdot + } + {\text{AH}} \to {\text{ABTS}} + {\text{A}}^{\cdot} + {\text{H}}^{ + } $$

The ABTS-radical-scavenging activity was affected by the extraction temperature in this study (Fig. 4B), increasing gradually as the temperature of the subcritical water increased, and especially from 170 to 190 °C. The highest activity of 90.8 ± 4.3% was observed at 200 °C for 20 min, which was the highest temperature possible in the laboratory-scale system. The ABTS-radical antioxidant activity also increased with the extraction time, and was highest at 200 °C for 20 min. The results obtained in the ABTS^·+^ assay were strongly correlated with those for the phenolics and flavonoids contents. At higher SWE temperatures of around 200 °C, extracts could be selectively analyzed for antioxidant activity.

The ABTS-radical-scavenging activity at an extraction temperature of 220 °C was optimal (96.0 ± 2.9%) when using pilot-scale SWE (Table 1). The ABTS-radical-scavenging ability was decreased as the temperature increasing up to 240 °C. It is considered that many bioactive compounds are unstable and destroyed at a high temperature of 240 °C.

### FRAP assay

The FRAP assay is an electron-transfer-based method^32^ involving a single-electron transfer reaction between an antioxidant, a single electron donor, and Fe (TPTZ)_2_ (III)^33^.

4 $$ {\text{Fe }}\left( {{\text{TPTZ}}} \right)_{2} \left( {{\text{III}}} \right) + {\text{AH}} \to {\text{Fe }}\left( {{\text{TPTZ}}} \right)_{2} \left( {{\text{II}}} \right) + {\text{AH}}^{\cdot + } $$

The FRAP antioxidant activity showed a similar tendency to the results of the ABTS assay of antioxidant activity, with the highest activity of 454.4 ± 4.9 mg TE/g occurring at 200 °C for 20 min when using laboratory-scale SWE (Fig. 4C).

The FRAP assay indicated that an extraction temperature of 220 °C was optimal (662.4 ± 17.2 mg TE/g) when using pilot-scale SWE (Table 1). The FRAP antioxidant activity was also decreased as the temperature increasing up to 240 °C.

### Correlations between antioxidant activities and subcritical water conditions

There were significant correlations between the values obtained using all of the measurement methods. The DPPH and ABTS assays are mixed methods using hydrogen-atom transfer and single-electron transfer, while the FRAP assay is based on single-electron transfer. The other antioxidant activities were significantly correlated with the results of the FRAP assay, indicating that the single-electron transfer mechanism acts on the antioxidant activity of extracts. The antioxidant activity was strongly correlated with the total phenolics content and the total flavonoids content, suggesting that phenolics and flavonoids in SWE extracts greatly affect the antioxidant activity. Because phenolics act as antioxidants via hydrogen-atom transfer and single-electron transfer^34^, the presence of aromatic structures and hydroxyl substituents plays a key role in the antioxidant activity of phenolics^35^. Flavonoids also act as antioxidants due to their low reduction potential^36^. The correlation coefficients for the total phenolics and flavonoids contents were higher for the ABTS assay (0.952 and 0.842, respectively) and the FRAP assay (0.853 and 0.709) than for the DPPH assay (0.633 and 0.717) of significant correlation at *p* < 0.01. These results are similar to those^21^.

### Comparison of activities among extraction solvents

The optimum condition was the OJ extracts obtained at 220 °C for 15 min using SWE, which was higher than when using conventional extraction methods using ethanol and methanol as solvents (Table 2). The phenolics and flavonoids contents of extracts obtained by SWE were 3.7- to 11.5-fold and 1.8- to 3.2-fold higher than those obtained when using extraction solvents with methanol and ethanol, respectively, for 2 h. Antioxidant activities of SWE extracts including DPPH, ABTS, and FRAP were also higher than when using the organic solvents methanol and ethanol at 25 °C and 60 °C, respectively, for 2 h.

**Table 2:** Comparison of total phenolics contents, flavonoids contents, and antioxidant activities of OJ among extraction solvents. Means in a row followed by same superscript letters are not significantly different according to Duncan’s test at *p* < 0.05.

	Extraction solvent, temperature, and time				
	Subcritical water 220 °C and 15 min	Methanol 25 °C and 2 h	Ethanol 25 °C and 2 h	Methanol 60 °C and 2 h	Ethanol 60 °C and 2 h
Total phenolics content (mg GAE/g)	39.9 ± 4.1^c^	6.87 ± 0.4^ab^	3.5 ± 0.3^a^	10.7 ± 1.3^b^	8.0 ± 2.6^b^
Total flavonoids content (mg QE/g)	11.4 ± 0.6^c^	4.03 ± 0.2^a^	3.6 ± 0.8^a^	5.8 ± 1.2^b^	6.1 ± 1.0^b^
DPPH-radical-scavenging ability (%)	90.3 ± 2.2^c^	89.57 ± 1.7^bc^	70.9 ± 4.3^a^	89.7 ± 0.3^bc^	81.0 ± 9.2^b^
ABTS-radical-scavenging ability (%)	96.0 ± 2.9^d^	57.70 ± 3.0^b^	19.6 ± 0.8^a^	78.7 ± 9.9^c^	57.0 ± 11.3^b^
FRAP antioxidant activity (mg TE/g)	662.4 ± 17.2^c^	328.9 ± 11.0^b^	130.6 ± 18.0^a^	318.7 ± 6.1^b^	177.1 ± 7.4^a^

The data are mean ± SD values (*n* = 3).

The results obtained in this study demonstrate that SWE is a highly efficient and rapid method for extracting bioactive compounds such as phenolics and flavonoids, and thus that subcritical water could be an excellent environmentally friendly alternative to using organic solvents such as methanol and ethanol for extracting antioxidant compounds.

## Conclusions

The phenolics contents, flavonoids contents, triterpene saponins, and antioxidant activities of SWE extracts were strongly affected by both the extraction temperature and extraction time. The overall results demonstrated that the extracts obtained from OJ in SWE at 220 °C for 15 min had the highest phenolics, flavonoids, and antioxidant activity, and also compared to using conventional extraction methods (using ethanol and methanol as solvents). Triterpene saponins were observed only in SWE condition at 220 °C, 15 min. At higher temperatures above 240 °C, most of bioactive compounds appeared to be unstable and destroyed. SWE is eco-friendly a rapid method for recovering antioxidant compounds and would be a useful technology for the production of bioactive compounds.

## References

[1] Lee JH, Lee SJ, Park S, Kim HK, Jeong WY, Choi JY, Sung NJ, Lee WS, Lim CS, Kim GS, Shin SC (2011). Characterisation of flavonoids in *Orostachys japonicus* A. Berger using HPLC-MS/MS: contribution to the overall antioxidant effect. Food Chem..

[2] Heim KE, Tagliaferro AR, Bobilya DJ (2002). Flavonoid antioxidants: chemistry, metabolism and structure-activity relationships. J. Nutr. Biochem..

[3] Caoab G, Sofica E, Prior RL (1997). Antioxidant and prooxidant behavior of flavonoids: structure-activity relationships. Free Radical Biol. Med..

[4] Zhang XF, Yang SL, Han YY, Zhao L, Lu GL, Xia T, Gao LP (2014). Qualitative and quantitative analysis of triterpene saponins from tea seed pomace (*Camellia oleifera* Abel) and their activities against bacteria and fungi. Molecules.

[5] Kim JH, Han SY, Kwon JH, Lee DS (2020). *Orostachys japonicus* ethyl acetate fraction suppresses MRSA biofilm formation. Asian Pac. J. Trop. Med..

[6] Kim SM, Park JH, Boo HO, Song SG, Park HY (2017). In vitro comparision of biological activities of solvent fraction extracts from *Orostachys japonicus*. Korean J. Plant Res..

[7] Park HJ, Yang HJ, Kim KH, Kim SH (2015). Aqueous extract of *Orostachys japonicus* A. Berger exerts immunostimulatory activity in RAW 264.7 macrophages. J. Ethnopharmacol..

[8] Ko MJ, Cheigh CI, Chung MS (2014). Relationship analysis between flavonoids structure and subcritical water extraction (SWE). Food Chem..

[9] Ayala RS, De Castro ML (2001). Continuous subcritical water extraction as a useful tool for isolation of edible essential oils. Food Chem..

[10] Teo CC, Tan SN, Yong JWH, Hew CS, Ong ES (2010). Pressurized hot water extraction (PHWE). J. Chromatogr. A.

[11] Ko MJ, Cheigh CI, Cho SW, Chung MS (2011). Subcritical water extraction of flavonol quercetin from onion skin. J. Food Eng..

[12] Yilmaz Y, Toledo RT (2004). Major flavonoids in grape seeds and skins: antioxidant capacity of catechin, epicatechin, and gallic aicd. J. Agric. Food Chem..

[13] Cheigh CI, Yoo SY, Ko MJ, Chang PS, Chung MS (2015). Extraction characteristics of subcritical water depending on the number of hydroxyl group in flavonols. Food Chem..

[14] Chang CC, Yang MH, Wen HM, Chern JC (2002). Estimation of total flavonoid content in propolis by two complementary colorimetric methods. J. Food Drug Anal..

[15] Bondet V, Brand-Williams W, Berset C (1997). Kinetics and mechanisms of antioxidant activity using the DPPH free radical method. LWT Food Sci. Technol..

[16] Benzie IF, Strain JJ (1996). The ferric reducing ability of plasma (FRAP) as a measure of “antioxidant power”: the FRAP assay. Anal. Biochem..

[17] Wang D, Huo R, Cui C, Gao Q, Zong J, Wang Y, Sun Y, Hou R (2019). Anticancer activity and mechanism of total saponins from the residual seed cake of *Camellia oleifera* Abel. in hepatoma-22 tumor-bearing mice. Food Funct..

[18] Carr AG, Mammucari R, Foster NR (2011). A review of subcritical water as a solvent and its utilisation for the processing of hydrophobic organic compounds. Chem. Eng. J..

[19] Costa MN, Muniz MA, Negrao CA, Costa CE, Lamarao ML, Morais L, Junior JO, Costa RM (2014). Characterization of pentaclethra macroloba oil. J. Therm. Anal. Calorim..

[20] Wang Y, Ke L, Yang Q, Peng Y, Hu Y, Dai L, Jiang L, Wu Q, Liu Y, Ruan R, Fu G (2019). Biorefinery process for production of bioactive compounds and bio-oil from *Camellia oleifera* shell. Int. J. Agric. Biol. Eng..

[21] Ko MJ, Lee JH, Nam HH, Chung MS (2017). Subcritical water extraction of phytochemicals from *Phlomis umbrosa* Turcz. Innov. Food Sci. Emerg..

[22] Ho CHL, Cacace JE, Mazza G (2007). Extraction of lignans, proteins and carbohydrates from flaxseed meal with pressurized low polarity water. LWT Food Sci. Technol..

[23] Kumar MSY, Dutta R, Prasad D, Misra K (2011). Subcritical water extraction of antioxidant compounds from Seabuckthorn (*Hippophae rhamnoides*) leaves for the comparative evaluation of antioxidant activity. Food Chem..

[24] Smith RM (2002). Extractions with superheated water. J. Chromatogr. A.

[25] Park JG, Park JC, Hur JM, Park SJ, Choi DR, Shin DY, Park KY, Cho HW, Kim MS (2000). Phenolic compounds from *Orostachys japonicus* having anti-HIV-1 protease activity. Nat. Prod. Sci..

[26] Andersson T, Hartonen K, Hyötyläinen T, Riekkola M (2003). Stability of polycyclic aromatic hydrocarbons in pressurized hot water. The Analyst.

[27] Ko MJ, Kwon HL, Chung MS (2016). Pilot-scale subcritical water extraction of flavonoids from satsuma mandarin (*Citrus unshiu* Markovich) peel. Innov. Food Sci. Emerg..

[28] Kwon HL, Chung MS (2015). Pilot-scale subcritical solvent extraction of curcuminoids from *Curcuma long* L.. Food Chem..

[29] Liang N, Kitts DD (2014). Antioxidant property of coffee components: assessment of methods that define mechanisms of action. Molecules.

[30] Miller NJ, Rice-Evans CA (1997). Factors influencing the antioxidant activity determined by the ABTS^·+^ radical cation assay. Free Radic. Res..

[31] Aliaga C, Lissi EA (2000). Reactions of the radical cation derived from 2,2'-azinobis (3-ethylbenzothiazoline-6-sulfonic acid) (ABTS^·+^) with amino acids. Kinetics and mechanism. Can. J. Chem..

[32] Yoo KM, Kim DO, Lee CY (2007). Evaluation of different methods of antioxidant measurement. Food Sci. Biotechnol..

[33] Ou B, Huang D, Hampsch-Woodill M, Flanagan JA, Deemer EK (2002). Analysis of antioxidant activities of common vegetables employing oxygen radical absorbance capacity (ORAC) and ferric reducing antioxidant power (FRAP) assays: a comparative study. J. Agric. Food Chem..

[34] Rice-Evans CA, Miller NJ, Paganga G (1996). Structure-antioxidant activity relationships of flavonoids and phenolic acids. Free Radic. Biol. Med..

[35] Villaño D, Fernández-Pachón MS, Moyá ML, Troncoso AM, García-Parrilla MC (2007). Radical scavenging ability of polyphenolic compounds towards DPPH free radical. Talanta.

[36] Pietta PG (2000). Flavonoids as antioxidants. J. Nat. Prod..

